# Serial Non-Invasive Myocardial Work Measurements for Patient Risk Stratification and Early Detection of Cancer Therapeutics-Related Cardiac Dysfunction in Breast Cancer Patients: A Single-Centre Observational Study

**DOI:** 10.3390/jcm12041652

**Published:** 2023-02-19

**Authors:** Ana Moya, Dimitri Buytaert, Monika Beles, Pasquale Paolisso, Jürgen Duchenne, Greet Huygh, Ciska Langmans, Adelheid Roelstraete, Sofie Verstreken, Marc Goethals, Riet Dierckx, Jozef Bartunek, Martin Penicka, Guy Van Camp, Ward A. Heggermont, Marc Vanderheyden

**Affiliations:** 1 Cardiovascular Research Center, OLV Hospital, Moorselbaan 164, B-9300 Aalst, Belgium; 2 CardioPath PhD Program, Department of Advanced Biomedical Sciences, Cardiovascular Pathophysiology and Therapeutics, University of Naples Federico II, 80131 Naples, Italy; 3 Department of Cardiovascular Sciences, KU Leuven, Herestraat 49, B-3000 Leuven, Belgium; 4 Department of Oncology and Radiotherapy, OLV Hospital, Moorselbaan 164, B-9300 Aalst, Belgium

**Keywords:** Myocardial Work, Cancer Therapeutics-Related Cardiac Dysfunction, pressure–strain loops, anthracyclines, cardiotoxicity, speckle tracking

## Abstract

Serial transthoracic echocardiographic (TTE) assessment of LVEF and GLS are the gold standard in screening Cancer Therapeutics-Related Cardiac Dysfunction (CTRCD). Non-invasive left-ventricle (LV) pressure–strain loop (PSL) emerged as a novel method to quantify Myocardial Work (MW). This study aims to describe the temporal changes and longitudinal trajectories of MW indices during cardiotoxic treatment. We included 50 breast cancer patients with normal LV function referred for anthracycline therapy w/wo Trastuzumab. Medical therapy, clinical and echocardiographic data were recorded before and 3, 6, and 12 months after initiation of the chemotherapy. MW indices were calculated through PSL analysis. According to ESC guidelines, mild and moderated CTRCD was detected in 10 and 9 patients, respectively (20% CTRCD_mild_, 18% CTRCD_mod_), while 31 patients remained free of CTRCD (62% CTRCD_neg_). Prior to chemotherapy MWI, MWE and CW were significantly lower in CTRCD_mod_ than in CTRCD_neg_ and CTRCD_mild_. Overt cardiac dysfunction in CTRCD_mod_ at 6 months was accompanied by significant worse values in MWI, MWE and WW compared to CTRCD_neg_ and CTRCD_mild_. MW features such as low baseline CW, especially when associated with a rise in WW at follow-up, may identify patients at risk for CTRCD. Additional studies are needed to explore the role of MW in CRTCD.

## 1. Introduction

Cardiotoxicity is a growing concern in patients referred for chemotherapy and anti-cancer drugs. This is in part related to the significantly prolonged survival of cancer patients in general but also because of the growth of the therapeutic armamentarium with the emergence of novel targeted cancer therapies. Several types of chemotherapeutics are related to cardiac dysfunction [1,2] which poses a management dilemma in balancing between a potentially life-saving anti-cancer treatment and the risk of Cancer Therapeutics-Related Cardiac Dysfunction (CTRCD) [3,4]. In the new ESC guidelines on cardio-oncology, the main cardiovascular (CV) risk factors are used to assess the risk for and monitor patients with CTRCD. Unfortunately, specific measurable CTRCD predictors are still lacking [5,6].

Echocardiography is the method of choice for cardiac function surveillance [6,7] due to its widespread use, accessibility and scientific foundation. In the same ESC guidelines, symptomatic heart failure (HF) is differentiated from asymptomatic HF in CTRCD. The severity of CTRCD in asymptomatic patients is determined by the decrease in the left ventricular ejection fraction (LVEF) in combination with relative changes in global longitudinal strain (GLS) and levels of serum cardiac biomarkers [6,8,9]. 

Nonetheless, all these indices have limitations and lack specificity in the surveillance throughout cancer treatment as it was proven in prospective follow-up studies [10,11]. Of note, GLS which can detect early changes in left-ventricle (LV) function and is recommended in the cardio-oncology guidelines, is influenced by blood pressure (BP) changes [9]. This afterload dependency will impact its accuracy even in the absence of any change in myocardial function. Non-invasive Myocardial Work (MW) indices derived from LV pressure–strain loops (PSL) can overcome this load-dependent limitation by incorporating estimated LV pressure in their equation [12]. This novel approach characterizes better LV function and allows to prognosticate adverse outcomes within various cardiac conditions [13,14,15]. In addition, the assessment of iso-volumetric contraction and relaxation as well as the ejection phase by MW indices, grants a more granular evaluation of the myocardial function [16,17]. Unfortunately, data validating MW in patients receiving chemotherapeutics are still scarce [18,19,20,21].

Therefore, this observational longitudinal follow-up study was set up to investigate the temporal changes and longitudinal trajectories of MW indices during cancer treatment and assess the potential role of serial MW analysis in patient risk stratification and early detection of CTRCD.

## 2. Materials and Methods

### 2.1. Study Population

In a prospective cohort, we recruited 50 breast cancer patients receiving sequential therapy with anthracyclines w/wo Trastuzumab between April 2016 and September 2020. All patients followed a similar anthracycline and taxane treatment regimen consisting of a combination of 4 cycles of Epirubicin and Cyclophosphamide every 2 or 3 weeks followed by 12 weekly doses of Paclitaxel. In addition, 33 HER2+ breast cancer patients received Trastuzumab every three weeks with the first dose being administered jointly with the first dose of Paclitaxel. Patients with a history of coronary artery disease, valvular stenosis or regurgitation of more than moderate severity, persistent atrial fibrillation, episodes of heart failure (≥NYHA 2) before chemotherapy or depressed LV function (LVEF < 50% and GLS > −18%) at baseline were excluded [5]. Patient’s medical history and CV risk factors were collected at inclusion. Medical therapy, clinical parameters and echocardiographic data were recorded before onset of the chemotherapy (baseline) and during follow-up (at 3, 6, and 12 months).

### 2.2. CTRCD Definition

CTRCD was defined using LVEF and GLS according to the recent ESC guidelines. Patients were categorized as having no CTRCD (CTRCD_neg_) with a LVEF ≥ 50% at follow-up and no relative decline in GLS > 15% from baseline, mild CTRCD (CTRCD_mild_) with a LVEF ≥ 50% and a relative decline in GLS > 15% from baseline, moderate CTRCD (CTRCD_mod_) with a LVEF < 50% together with a relative decline in GLS > 15% from baseline and severe CTRCD with a LVEF < 40% [6]. As none of the patients met the threshold for severe CTRCD, only patients with no, mild and moderate CTRCD were included in the analysis. Patients were grouped for analysis regardless the time of CTRCD onset.

### 2.3. Transthoracic Echocardiography

All patients underwent serial standard greyscale and Doppler transthoracic echocardiograms at baseline and at 3, 6, and 12 months using GE Vivid 7 and 9 ultrasound systems (General Electric Vingmed Ultrasound, Horten, Norway) equipped with a 5-MHz multifrequency transducer. Dedicated apical 2- and 4-chamber images were obtained for measurement of bi-plane LVEF (Simpson method). For measurements of GLS, optimized 2-, 3- and 4-chamber apical view images of the LV were obtained for 3 cardiac cycles at high frame rates (55–75 frame per second) and were stored digitally for off-line analysis using dedicated software (EchoPac, GE Vingmed Ultrasound, Horten, Norway).

### 2.4. Speckle Tracking Imaging

Semi-automated 2D speckle tracking was applied to the three apical views to assess 2D-GLS and to obtain the LV strain bull-eye with the segmental strain values. Myocardium contour and region of interest (ROI) width were manually adjusted if necessary by the operator according to patient anatomy. The timing of aortic and mitral valve events was visually determined at the apical 3-chamber view. After measuring the peak negative value on the strain curve, GLS was calculated as the average from all LV segments interrogated [9,22].

### 2.5. MW Assessment

MW analysis was performed off-line and was not intended to guide patients’ therapy during follow-up. During post-processing strain, non-invasive BP, measured at the brachial artery at the time of the examination, and valve events were integrated with an automated dedicated software tool. This software package constructs a non-invasive LV pressure curve, adjusted according to the duration of isovolumic and ejection phases defined by valvular timing events [23]. Strain and pressure data were synchronized using the onset of R-wave on the ECG. MWI, MWE, global CW and global WW were recorded. MWI, derived from the area of the LV PSL, reflects the total work calculated from mitral valve closure to mitral valve opening, CW is the work performed by the LV contributing to LV ejection during systole, WW is the work performed by the LV that does not contribute to LV ejection. MWE is defined by CW divided by the sum of CW and WW. Global values were obtained as average from all myocardial segments [12].

### 2.6. Reproducibility

The intra- and interobserver variability for GLS and MW are reported in Table A1 Appendix A. Briefly, ten patients were randomly selected, and repeated measures were performed by two independent observers (expert echocardiographers) blinded to the patient’s clinical data and each other’s results. Intraobserver variability was performed by the sonographer on off-line data at different points in time. Interobserver variability was performed by repeating measurements from the same images by the 2 sonographers. Intra- and interobserver variability were calculated by intraclass coefficient (ICC) and the standard error of measurements.

### 2.7. Statistics

Descriptive data are reported as the mean ± SD for normally distributed continuous variables. Normality testing was performed by graphical analysis with histograms and QQ-plots. Differences between groups were compared using the one-way ANOVA test for continuous variables and Pearson’s χ^2^ test for categorical variables. Additionally, CTRCD_neg_ and CTRCD_mild_ were grouped together for comparison with CTRCD_mod_ using an independent 2-tailed T-test. The predictive value for moderate CTRCD of MW and GLS indices was analyzed using a random effects logistic regression model with random slope. For a clinical meaningful interpretation of the fixed effects of the regression model we rescaled the predictor variables with 10^−3^ for MWI and CW and with 10^−1^ for MWE. Odds ratios and 95% CI were reported. For all tests, a *p*-value below 0.05 was considered statistically significant. All statistics analysis were performed using SPSS for Windows Version 25 (SPSS, IBM headquarters, Armonk, NY, USA).

## 3. Results

### 3.1. Baseline Patients’ Characteristics

Between April 2016 and September 2020, 50 breast cancer patients (mean age 56 ± 12 years) were included in this study and were followed for at least 1 year after the start of their chemotherapy. During follow-up, moderated CTRCD was detected in 9 patients (18% CTRCD_mod_), occurring at 6 and 12 months in 5 and 4 patients, respectively, mild CTRCD was observed in 10 patients (20% CTRCD_mild_) occurring at 3, 6 and 12 months in 3, 4 and 3 patients, respectively, and 31 patients remained free of CTRCD (62%, CTRCD_neg_). None of the patients developed severe CTRCD at follow-up.

Baseline patients’ characteristics are summarized in Table 1. No significant differences in CV risk factors, therapy or clinical parameters were noted between the different groups.

### 3.2. Temporal Changes in LV Systolic Function in Patients with/without Cardiotoxicity

Echocardiographic data are presented in Table 2. Before the onset of cancer therapy, all patients presented with normal LV systolic and diastolic function. Interestingly, although baseline GLS was within normal limits in the three cohorts, those in the CTRCD_mod_ group presented with an already significantly lower GLS at baseline compared to the CTRCD_neg_ and CTRCD_mild_ groups.

**Figure 1:**
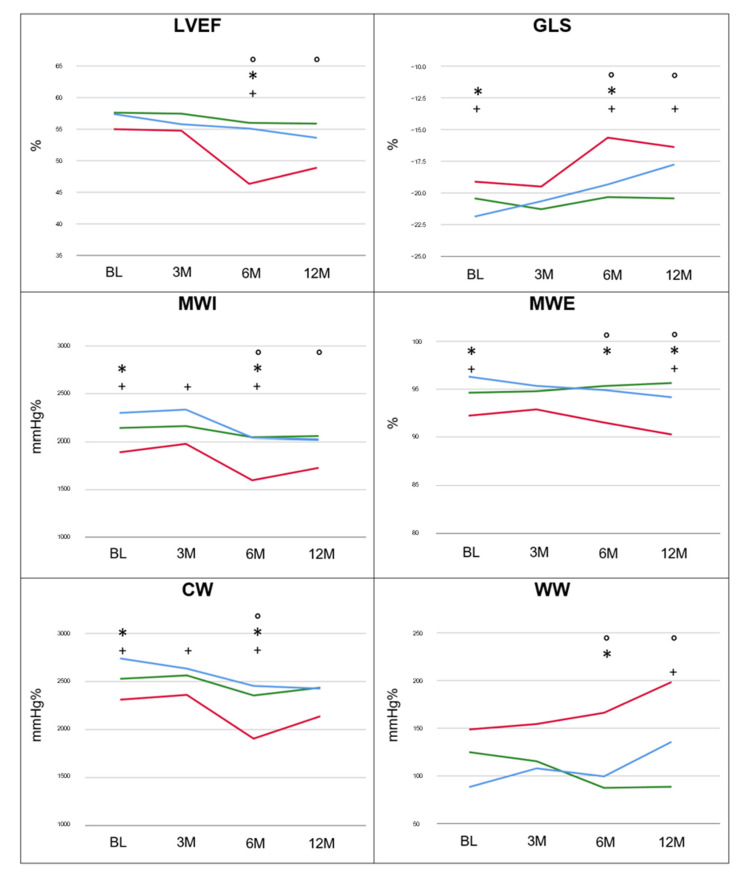
Temporal changes and trajectories of LVEF, GLS and MW indices in CTRCD_neg_ (green), CTRCD_mild_ (blue) and CTRCD_mod_ (red) group. ^°^ CTRCD_mod_ vs. CTRCD_neg_; * CTRCD_mod_ vs. CTRCD_mild_; ^+^ CTRCD_mod_ vs. CTRCD_neg + mild_; *p* ≤ 0.05.

In the CTRCD_neg_ and CTRCD_mild_ groups, the LVEF remained unchanged at follow-up, whereas, in the CTRCD_mod_ group, LVEF at 6 months was significantly lower compared to CTRCD_neg_ and CTRCD_mild_ patients. At 12 months, LV dysfunction persisted in the CTRCD_mod_ group (Table 2, Figure 1).

By definition, GLS remained unchanged at follow-up in CTRCD_neg_ patients, whereas it significantly worsened in CTRCD_mild_ and CTRCD_mod_ (Table 2). In this last group, the GLS at 6 and 12 months was significantly impaired compared to the other groups. Interestingly, the deterioration of GLS in the CTRCD_mild_ group was accompanied by a significantly higher afterload at 12 months compared to the other groups (Table 3).

### 3.3. Temporal Changes and Longitudinal Trajectories of MW in Patients with/without Cardiotoxicity

Temporal changes in MW indices are summarized in Table 3 together with BP values as estimation of afterload variation. Longitudinal trajectories of MW are graphically presented in Figure 1 together with the concurrent progression of LVEF and GLS. Baseline MWI, MWE and CW were significantly lower in the CTRCD_mod_ group compared to the CTRCD_neg_ and CTRCD_mild_ groups. However, baseline WW was similar in all groups. For all MW indices, the CTRCD_mod_ group showed persistent worse values during follow-up.

Despite equivalent values between the different cohorts at 3 months, MW indices remarkably deteriorated at 6 months and were significantly worse in the CTRCD_mod_ group compared to the CTRCD_mild_ and CTRCD_neg_ groups. For MWI, WW and MWE this intergroup difference remained significant at 12 months.

**Figure 2:**
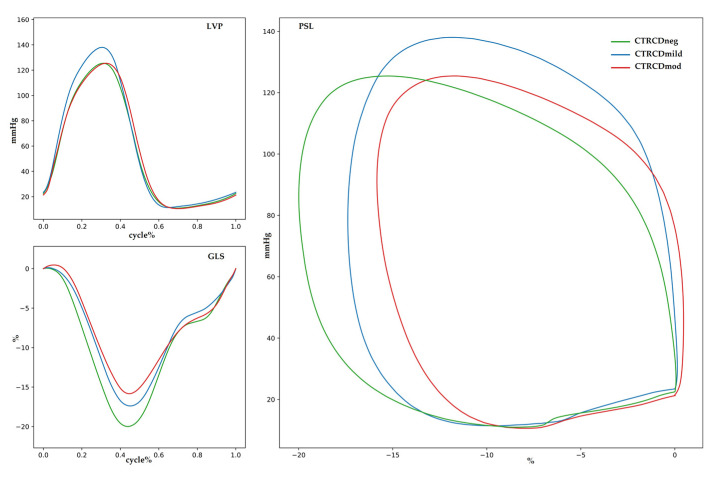
PSL analysis for CTRCD_neg_ (green), CTRCD_mild_ (blue) and CTRCD_mod_ (red) at 12 M follow-up.

**Figure 3:**
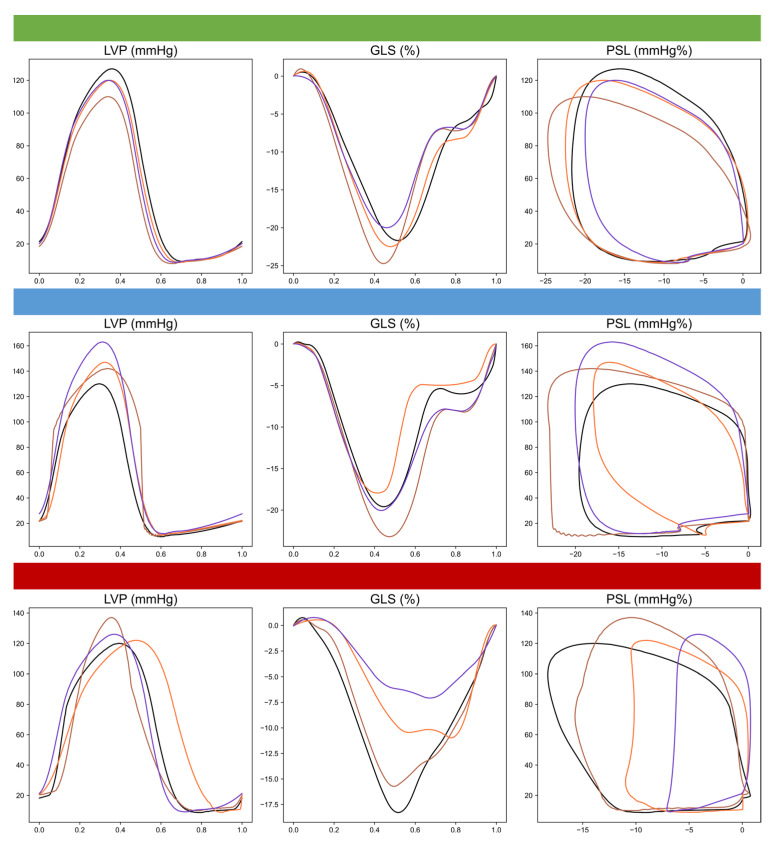
An illustrative example of temporal changes in LVP, GLS and MWI, graphically represented by the PSL area, in three different patients without and with mild or moderate CTRCD. Black: BL, brown: 3 M, orange: 6 M, purple: 12 M.

The trajectories of MWI, MWE, CW and WW were significantly different between patients with and without CTRCD as well as between those with mild and moderate CTRCD (Figure 1). This is visually expressed in the differences in PSL area at 12 months follow-up (Figure 2) between the three groups. Whereas CTRCD_neg_ and CTRCD_mild_ had similar MWI values and PSL area at 12 months, the narrower and more elongated PSL in CTRCD_mild_ suggests a physiological decrease in GLS due to increased afterload but without myocardial impairment. In contrast, the lower MWI value in CTRCD_mod_ is consistent with the smaller PSL area. Finally, an illustrative example of the different temporal changes in MWI in an individual patient, represented by the PSL area, is shown in Figure 3.

### 3.4. Predictive Value of MW Indices and GLS for CTRCD

The random effect logistic regression model demonstrated that MWI, MWE and CW were significant univariate predictors for moderate CTRCD. By contrast, GLS and WW were unable to predict moderate CTRCD. The odds ratio and confidence interval for fixed effects are summarized in Table 4. 

## 4. Discussion

Our observational single-center study demonstrates that in a female population with breast cancer undergoing treatment with anthracyclines, the assessment of non-invasive MW by LV PSL analysis helps to estimate patient’s risk and detect CTRCD. We demonstrated that patients with previous normal GLS but lower CW were at higher risk for developing CTRCD. In addition to lower baseline CW, patients with moderate CTRCD were characterized by a significant higher WW at follow-up. We conclude that MW assessment may be an additional confirmatory marker for patient risk stratification and detection of subsequent CTRCD. Therefore, further prospective studies addressing its use in breast cancer patients are warranted. 

### 4.1. Surveillance and Precision in CTRCD Diagnosis

As cardiotoxicity may become among the main determinants of poor quality of life and mortality in oncologic patients, the early detection of myocardial tissue damage related to cancer therapy remains of utmost importance. Currently, cardiac surveillance is guided by serial 2D echocardiographic evaluation of LVEF which is affected by several limitations including load dependency, its substantial variability and the need of geometric assumptions for its calculation. As an alternative, 2D-GLS has shown optimal feasibility and reproducibility and its relative change precedes LVEF reduction during chemotherapy [24]. However, also this technique is hampered by limitations such as the intervendor variability, technical requirements intrinsic to the strain technology and its load dependency [25]. In our study, all patients started anti-cancer therapy with intravenous anthracyclines administration every 3 weeks and completed this part of the treatment after 3 months. As anthracyclines provoke a cumulative dose-dependent cardiotoxic damage with irreversible cellular necrosis (Type 1 CTRCD), changes in LVEF and GLS are usually not detected before 3 months follow-up [19]. This is in line with our results where the LV systolic function in the CTRCD_mild_ and CTRCD_mod_ groups was normal at 3 months and an evident deterioration was detected at 6 months follow-up in the CTRCD_mod_ group when both LVEF and GLS were impaired compared to CTRCD_neg_ and CTRCD_mild_. This relatively late detection of LV dysfunction may slow down the timely initiation of cardioprotective treatment and indicates the need for earlier detection of subtle changes in LV function to surveil hemodynamic deterioration.

### 4.2. The Role of MW in CTRCD

To overcome the load dependency limitation of GLS, Russel et al. developed a non-invasive method for MW assessment which implements the patient’s loading condition at the time of examination and offers a more complete picture of the LV function by quantifying both constructive as well as wasted work [26]. Although MW indices, particularly MWI and CW, have diagnostic and prognostic value in a variety of cardiovascular conditions [13,14,15], its role in patients receiving chemotherapy is limited.

In our results, baseline quantification of MW indices allowed to identify patients at higher risk of developing CTRCD. Interestingly, those with moderate CTRCD differentiated from the other groups by a lower MWE and CW at baseline. Moreover, we demonstrated that MWI, MWE and CW (and not WW and GLS) were able to predict moderate CTRCD. This in line with previous observations that showed a lower MWE at baseline in patients who developed CTRCD during follow-up [27]. As MWE is related to variations in both CW and WW with CW reflecting the Myocardial Work that contributes to cardiac output and WW the work that does not contribute to it, changes in each of these parameters could account for this observation. In contrast to the work of Calvillo-Argüelles et al., we demonstrated that not a higher WW but rather lower CW was responsible for the depressed MWE at baseline in patients with moderated CTRCD during follow-up. This may suggest there exists a distinct phenotype with a higher susceptibility for developing CTRCD, characterized by an inherent less efficient LV performance due to lower contractile capacity [27]. Therefore, MW features such as low baseline CW, especially when associated with a rise in WW during follow-up, may identify patients at risk for CTRCD.

### 4.3. Limitations

This study presents some limitations. First, MW assessment is a relatively new and unexplored method especially in cancer patients where clear and robust reference values are still lacking. The adoption of MW assessment in routine practice requires the commitment of echocardiography laboratories and professionals’ training. As experience is important for precision in strain measurements, sites adopting this approach should promote minimal annual volumes to maintain competency. Second, this advanced and sophisticated analysis requires high quality images that are not easy to acquire in the daily practice. This study was also limited by its small sample size and single-center design which hampers statistical analysis to explore the role of MW indices in the multivariate context. Certainly, larger scale studies are needed for further validation of these results and to establish the clinical utility of MW in cancer patient surveillance.

In contrast with previous studies [28,29,30], we did not collect data on the effect of cardioprotective treatment as our analysis was observational and not intended to guide medical therapy. Additionally, we categorized patients according to the first ESC guidelines on cardio-oncology which were published only after patients’ data were collected. Finally, the addition of cardiac biomarkers to the analysis would definitely have enriched our results and longer follow-up would be of interest to explore the long-lasting cardiac effects of chemotherapy.

## 5. Conclusions

In women with breast cancer receiving treatment with anthracycline w/wo Trastuzumab, lower CW at baseline as well as higher WW during follow-up identifies a subgroup of patients at risk for developing CTRCD irrespectively of LVEF and GLS. Our findings point out the usefulness of non-invasive MW assessment as a sophisticated tool for risk stratification previous to cancer treatment as well as clinical surveillance of cardiac function during follow-up. These promising results warrant additional studies to explore the role of MW for prediction and early detection of CRTCD. Similar to strain-guided initiation of cardioprotective therapy in patients at risk for CTRCD, studies exploring the role of a MW-guided approach in this patient population are needed.

## Figures and Tables

**Table 1:** Baseline patients’ characteristics and medical therapy.

	CTRCD_neg_	CTRCD_mild_	CTRCD_mod_	*p*-Value	TOTAL
N (%)	31 (62)	10 (20)	9 (18)	-	50
Age, yo (mean ± SD)	56 ± 13	53 ± 11	59 ± 12	0.49	56 ± 12
Cardiovascular risk factors					
Diabetes mellitus, *n* (%)	2 (6.5)	0	0	0.53	2 (4)
Arterial hypertension, *n* (%)	6 (19.4)	2 (20)	1 (11.1)	0.84	9 (18)
Hyperlipidemia, *n* (%)	5 (16.1)	1 (10)	0	0.41	6 (12)
Obesity, *n* (%)	5 (16.1)	0	1 (11.1)	0.41	6 (12)
Smoking, *n* (%)	3 (9.7)	1 (10)	0	0.58	4 (8)
Alcohol, *n* (%)	0	0	0	0.87	0
Family history of CVD, *n* (%)	2 (6.5)	1 (10)	1 (11.1)	0.34	4 (8)
Medication					
Beta blocker, *n* (%)	3 (9.7)	1 (10)	1 (11.1)	0.99	5 (10)
ACE-I or ARB, *n* (%)	3 (9.7)	0	0	0.38	3 (6)
Cardiotoxic drugs					
Trastuzumab, *n* (%)	18 (58.1)	8 (80)	7 (77.8)	0.34	33 (66)
Epirubicin, cum. dose mg (mean ± SD)	574 ± 108	622 ± 94	598 ± 61	0.39	588 ± 99
Clinical parameters					
BMI (mean ± SD)	24.9 ± 4.1	25.9 ± 4.1	23.6 ± 2.2	0.50	25.0 ± 4
HR, bpm (mean ± SD)	75 ± 10	73 ± 9	73 ± 11	0.86	74 ± 10
QTc (mean ± SD)	422 ± 21	444 ± 34	435 ± 34	0.17	429 ± 27
QRS width, ms (mean ± SD)	82 ± 18	89 ± 5	89 ± 6	0.47	87 ± 7

**Table 2:** Echocardiographic data for CTRCD_neg_, CTRCD_mild_ and CTRCD_mod_ at baseline (BL) and follow-up (3 M, 6 M, 12 M).

		BL			3 M			6 M			12 M		
		CTRCD_neg_	CTRCD_mild_	CTRCD_mod_	CTRCD_neg_	CTRCD_mild_	CTRCD_mod_	CTRCD_neg_	CTRCD_mild_	CTRCD_mod_	CTRCD_neg_	CTRCD_mild_	CTRCD_mod_
**LV dimensions**	LVEDD (mean ± SD, mm)	46 ± 4	46 ± 4	47 ± 4	44 ± 6	47 ± 5	45 ± 6	44 ± 5	44 ± 6	46 ± 7	45 ± 4	45 ± 4	46 ± 12
	LVESD (mean ± SD, mm)	30 ± 4	29 ± 6	32 ± 4	29 ± 6	33 ± 5	30 ± 6	30 ± 4	31 ± 6	33 ± 10	31 ± 7	32 ± 4	36 ± 11
	LVEDV (mean ± SD, mm)	91 ± 21	101 ± 21	95 ± 21	80 ± 31	104 ± 25 °	80 ± 33	88 ± 20	93 ± 29	99 ± 29	84 ± 18	99 ± 16	92 ± 39
	LVESV (mean ± SD, mm)	34 ± 10	34 ± 11	34 ± 10	34 ± 16	43 ± 16	36 ± 14	36 ± 10 *	39 ± 16	48 ± 22 ^°^	37 ± 10 *	44 ± 10	52 ± 32 °
	LV mass (mean ± SD, g)	129 ± 32	140 ± 24	131 ± 33	144 ± 50	146 ± 57	131 ± 39	133 ± 51	148 ± 37	137 ± 27	133 ± 38	135 ± 24	150 ± 70
	LV mass index (mean ± SD, g/m^2^)	75 ± 18	78 ± 12	72 ± 16	80 ± 22	77 ± 13	69 ± 7	73 ± 19	76 ± 20	78 ± 16	73 ± 15	75 ± 14	69 ± 31
**LV-SF**	LVEF (mean ± SD, %)	57.6 ± 4.3	57.4 ± 3.6	55 ± 4.5	57.5 ± 5.4	55.8 ± 4.4	54.8 ± 8.7	56.0 ± 4.6 *	55.1 ± 4.5 *	46.3 ± 4.5 ^+^	55.9 ± 4.7 *	53.6 ± 3.0	48.9 ± 10.1
	GLS (mean ± SD, %)	−20.4 ± 2.5	−21.9 ± 1.6 *	−19.1 ± 1.7 ^+^	−21.3 ± 2.6	−20.7 ± 3.6	−19.5 ± 3.1	−20.3 ± 2.0 *	−19.3 ± 2.3 *	−15.6 ± 3.2 ^+^	−20.4 ± 2.9 *	−17.8 ± 2.5	−16.4 ± 4.4 ^+^
**LV-DF**	E peak (mean ± SD, m/s)	0.64 ± 0.17	0.68 ± 0.11	0.67 ± 0.18	0.66 ± 0.16	0.70 ± 0.16	0.68 ± 0.12	0.63 ± 0.17	0.71 ± 0.21	0.57 ± 0.15	0.65 ± 0.14	0.72 ± 0.15	0.69 ± 0.17
	A peak (mean ± SD, m/s)	0.72 ± 0.17	0.67 ± 0.17	0.78 ± 0.26	0.71 ± 0.18	0.70 ± 0.12	0.80 ± 0.21	0.68 ± 0.15	0.70 ± 0.14	0.71 ± 0.16	0.67 ± 0.18	0.74 ± 0.07	0.80 ± 0.22
	E/A ratio (mean ± SD)	0.95 ± 0.35	1.06 ± 0.25	0.93 ± 0.31	0.98 ± 0.37	1.02 ± 0.28	0.92 ± 0.33	0.97 ± 0.30	1.05 ± 0.31	0.81 ± 0.19	1.03 ± 0.36	0.99 ± 0.24	0.93 ± 0.31
	e’ sept (mean ± SD, m/s)	0.07 ± 0.03	0.07 ± 0.02	0.9 ± 0.04	0.07 ± 0.02	0.10 ± 0.02 ^°^	0.08 ± 0.03	0.07 ± 0.02	0.08 ± 0.03	0.08 ± 0.03	0.08 ± 0.03	0.18 ± 0.25	0.08 ± 0.02
	e’ lat (mean ± SD, m/s)	0.10 ± 0.04	0.10 ± 0.03	0.11 ± 0.06	0.09 ± 0.003	0.12 ± 0.04	0.10 ± 0.04	0.09 ± 0.02	0.11 ± 0.02	0.11 ± 0.04	0.11 ± 0.04	0.12 ± 0.03	0.11 ± 0.02
	E/e’ (mean ± SD)	8.1 ± 2.8	7.9 ± 2.5	9.6 ± 1.2	8.0 ± 2.2	7.5 ± 2.1	8.5 ± 2.1	7.5 ± 1.9	7.0 ± 1.8	7.0 ± 3.5	7.5 ± 2.2	7.6 ± 1.7	7.4 ± 2.3
**RV and LA**	LAVI (mean ± SD, mL/m^2^)	25 ± 7	21 ± 6	25 ± 15	25 ± 8	35 ± 4 *^,°^	20 ± 4	27 ± 9	29 ± 5	23 ± 7	30 ± 11	24 ± 3 *	40 ± 10
	TAPSE (mean ± SD, mm)	22 ± 4	23 ± 4	23 ± 4	23 ± 3	23 ± 3	21 ± 2	22 ± 4	23 ± 4	22 ± 4	23 ± 4	22 ± 3	21 ± 4

* vs. CTRCD_mod_; ° vs. CTRCD_neg_; ^+^ vs. CTRCD_neg+mild_; *p* ≤ 0.05.

**Table 3:** Myocardial Work indices and blood pressure (afterload) for CTRCD_neg_, CTRCD_mild_ and CTRCD_mod_ at baseline (BL) and follow-up (3 M, 6 M, 12 M).

	BL			3 M			6 M			12 M		
	CTRCD_neg_	CTRCD_mild_	CTRCD_mod_	CTRCD_neg_	CTRCD_mild_	CTRCD_mod_	CTRCD_neg_	CTRCD_mild_	CTRCD_mod_	CTRCD_neg_	CTRCD_mild_	CTRCD_mod_
MWI (mean ± SD, mmHg%)	2139 ± 364	2297 ± 338 *	1892 ± 215 ^+^	2159 ± 437	2332 ± 652	1981 ± 162 ^+^	2042 ± 380 *	2038 ± 386 *	1598 ± 347 ^+^	2054 ± 267 *	2021 ± 407	1728 ± 578
CW (mean ± SD, mmHg%)	2527 ± 391	2740 ± 383 *	2310 ± 265 ^+^	2563 ± 498	2633 ± 608	2360 ± 170 ^+^	2354 ± 392 *	2453 ± 317 *	1905 ± 447 ^+^	2436 ± 310	2426 ± 459	2138 ± 670
WW (mean ± SD, mmHg%)	125 ± 90	88 ± 50	149 ± 86	115 ± 88	108 ± 47	154 ± 79	87 ± 31 *	100 ± 32 *	166 ± 153	89 ± 48 *	136 ± 90	198 ± 103 ^+^
MWE (mean ± SD, %)	95 ± 4	96 ± 2*	92 ± 5 ^+^	95 ± 3	95 ± 2	93 ± 4	95 ± 2 *	95 ± 1 *	92 ± 6	96 ± 2 *	94 ± 3 *	90 ± 5 ^+^
SBP (mean ± SD, mmHg)	131 ± 15	134 ± 12	130 ± 12	125 ± 16	135 ± 18	131 ± 14	128 ± 14	132 ± 17	128 ± 9	128 ± 10	143 ± 21*^,^°	126 ± 12
DBP (mean ± SD, mmHg)	78 ± 10	85 ± 12 °	80 ± 11	72 ± 10	85 ± 12 °	75 ± 18	76 ± 8	76 ± 11	76 ± 9	73 ± 8	86 ± 12 *^,^°	70 ± 6

* vs. CTRCD_mod_, ° vs. CTRCD_neg_, ^+^ vs. CTRCD_neg+mild_, *p* ≤ 0.050.

**Table 4:** Univariate random effect logistic regression model for Myocardial. Work indices and GLS.

	Odds Ratio	95% CI	*p*-Value
MWI × 10^−3^	0.89	0.82–0.96	0.01
MWE × 10^−1^	0.98	0.96–0.99	0.04
CW × 10^−3^	0.91	0.85–0.98	0.01
WW × 10^−2^	1.00	0.99–1.00	0.06
GLS	1.00	0.99–1.01	0.43

## Data Availability

Not applicable.

## Appendix A

**Table A1:** Intra- and interobserver variability.

	Intraobserver Variability			Interobserver Variability		
	ICC (95% CI)	Bias	Limits of Agreement	ICC (95% CI)	Bias	Limits of Agreement
LVEF, %	0.616 (−0.144–0.871)	2.07 ± 5.61	−8.93 to 13.06	0.598 (0.068–0.859)	−4.07 ± 6.25	−16.31 to 8.18
GLS, %	0.898 (0.700–0.965)	0.80 ± 1.86	−2.84 to 4.44	0.861 (0.600–0.953)	−0.61 ± 2.10	−4.72 to 3.50
MWI, mmHg%	0.932 (0.782–0.978)	−101.0 ± 198.9	−490.99 to 288.86	0.869 (0.601–0.956)	2.80 ± 267.48	−521.47 to 527.07
MWE, %	0.921 (0.758–0.974)	−1.07 ± 2.28	−5.54 to 3.41	0.886 (0.441–0.967)	1.87 ± 2.20	−2.44 to 6.18
CW, mmHg%	0.925 (0.735–0.976)	−150.00 ± 249.95	−639.91 to 339.91	0.925 (0.778–0.975)	23.13 ± 229.69	−427.06 to 473.32
WW, mmHg%	0.952 (0.862–0.984)	14.27 ± 43.62	−71.22 to 99.75	0.815 (0.298–0.943)	−60.53 ± 78.61	−214.61 to 93.54
